# Application of the BOPPPS-CBL model in electrocardiogram teaching for nursing students: a randomized comparison

**DOI:** 10.1186/s12909-023-04983-x

**Published:** 2023-12-21

**Authors:** Heling Wen, Wentao Xu, Fuli Chen, Xiaoyan Jiang, Rui Zhang, Jianhui Zeng, Lei Peng, Yu Chen

**Affiliations:** 1 Department of Cardiology, Sichuan Academy of Medical Science &Sichuan Provincial People’s Hospital, University of Electronic Science and Technology of China, Chengdu, 610072 China; 2 School of Medicine, University of Electronic Science and Technology of China, Chengdu, 610075 China; 3 Department of Cardiovascular Surgery, The Seventh People’s Hospital of Chengdu, Chengdu, China; 4 Department of Cardiology, The Affiliated Hospital of North Sichuan Medical College, Nanchong, China; 5 Department of Nephrology, Sichuan Academy of Medical Science &Sichuan Provincial People’s Hospital, University of Electronic Science and Technology of China, Chengdu, 610072 China

**Keywords:** BOPPPS model, Case-based learning, Lecture-based learning, ECG interpretation, Nurses

## Abstract

**Background/Aim:**

Interpreting an electrocardiogram (ECG) is a vital skill for nurses in cardiology. This study aimed to evaluate the efficacy of the bridge-in, objective, preassessment, participatory learning, post-assessment, and summary (BOPPPS) model, when combined with case-based learning (CBL), in enhancing nursing students’ ECG interpretation capabilities.

**Materials & methods:**

Nursing students were randomly divided into two groups: one utilizing the BOPPPS model combined with CBL (BOPPPS-CBL), and the other employing a traditional lecture-based learning (LBL) model. All participants underwent training and completed pre- and post-course quizzes.

**Results:**

The BOPPPS-CBL model significantly improved nursing students’ abilities in ECG interpretation compared to the traditional LBL model group. The BOPPPS-CBL model proved to be a comprehensive and effective method for enhancing students’ attitudes towards teaching and learning.

**Discussion:**

Our study demonstrated for the first time that the BOPPPS-CBL model is an innovative and effective method for promoting nurses’ accuracy in ECG interpretation. It highlights the potential of this approach as a superior alternative to traditional learning methods.

## Introduction

Electrocardiograph (ECG) is a crucial diagnostic tool for cardiovascular diseases, particularly for the rapid diagnosis of malignant arrhythmia and acute coronary syndrome [1]. In departments such as cardiology (including the cardiac care unit and cardiac catheterization room), ECG is vital for high-risk patients when making medical decisions. With advancements in science and technology, modern ECG machines are now equipped with ECG analysis software, and artificial intelligence’s deep learning is increasingly integrated into precise disease diagnosis [2, 3]. These technological advancements are enhancing the machine’s ability to recognize and interpret ECGs. Nurses, often the primary performers of ECG and the first to discover abnormalities, must possess correct ECG diagnosis skills, especially those working in cardiology, cardiac care units (CCU), ICUs, and emergency medicine departments [4]. Their ability to identify high-risk ECG changes in critically ill patients is crucial to the quality of medical care. Conducting effective training to enable nurses to make rapid and accurate interpretations of ECGs can reduce the underdiagnosis and misdiagnosis of severe cardiovascular diseases, thus initiating timely treatment [4].

Teaching ECG interpretation is challenging due to the abstract nature of the knowledge and theory students must master for its high-level application [5]. Although traditional lecture-based learning (LBL) is the most common method in nursing education and offers the advantage of disseminating core theoretical knowledge to large groups of students [6, 7], recent research has shown it to be ineffective in enhancing students’ reasoning abilities, especially for practical subjects like ECG interpretation [7]. The teacher-oriented nature of the LBL method fails to effectively translate knowledge into student understanding. Furthermore, the lack of diagnostic training opportunities during traditional ECG teaching may dampen students’ interest and enthusiasm, hindering significant improvements in their high-risk ECG interpretation skills. Thus, the LBL method might not be conducive to fostering the clinical reasoning and practice abilities of nurses in cardiology, calling for innovations in teaching strategy to better suit their needs.

The current nursing education landscape relies heavily on the traditional LBL method, with teachers dominating the teaching process. Although recent studies have highlighted various teaching mode reforms in medical and nurse education, student-oriented changes specific to ECG teaching have rarely been reported. An urgent need exists for innovative teaching strategies to enhance nurses’ ECG interpretation skills, especially for those at high risk of cardiovascular events.

The BOPPPS model, a pedagogical framework introduced in 1984 at the University of British Columbia, is designed to foster student engagement through its six-stage instructional process: bridge-in, objective, pre-assessment, participatory learning, post-assessment, and summary [8]. This student-centered approach significantly enhances classroom instruction efficacy, boasting advantages over traditional methods in areas such as course satisfaction, student-teacher interaction, and clinical analytical ability elevation of the students [8]. The success of BOPPPS model across various disciplines, coupled with its potential to ignite students’ learning enthusiasm, renders the BOPPPS model an exciting tool for future implementation.

Recent evidence suggests that the BOPPPS model, particularly when adapted into hybrid teaching models that combine other learning strategies, has positively impacted both teaching efficacy and academic achievement [9–11]. As the BOPPPS model continues to evolve to meet the dynamic demands of education, its broad application and proven effectiveness make it a promising candidate for wider educational adoption.

Case-based learning (CBL), another specialized learner-centered approach, engages students through real-world scenarios, promoting higher levels of cognition [12, 13]. Through group collaboration and problem-solving, CBL enhances understanding of key concepts and integrates theoretical knowledge with practical application [13–15]. This method not only deepens students’ learning but also increases satisfaction [16, 17].

ECG teaching is uniquely challenging for medical students, with no single method universally recognized as most effective [18]. Recognizing this challenge, a preliminary investigation was conducted to evaluate the BOPPPS-CBL model for ECG teaching among nursing students. This fusion of pedagogical techniques aims to enhance ECG interpretation and diagnostic skills, addressing an area of vital importance in the training and development of medical professionals.

## Methods

### Study design

This was a randomized controlled trial study conducted in a teaching and research hospital during the academic years 2020–2022, involving two groups, three ECG tests, and a quantitative questionnaire survey. A total of 109 nursing students participating in this study have graduated with either a Bachelor’s Degree from a medical university or an Associate Degree from a vocational high school, hailing from the cardiology departments of various non-teaching hospitals. This study was held when these nursing students were undergoing a year-long cardiology training program designed to equip them to work proficiently and compassionately as nurses in specialized settings such as cardiac catheterization labs or cardiac care units. These nursing students were then randomly divided into two groups using a digital randomization method: fifty-five were trained using the BOPPPS‑CBL model as an experimental group, while fifty-four were trained using the traditional LBL method as a control group. The study received approval from the ethics committee, and informed consent was obtained from each participant. Furthermore, the study was conducted in accordance with the Declaration of Helsinki (2013).

### Teaching implementation

#### The BOPPPS‑CBL model for ECG course

**Fig. 1:**
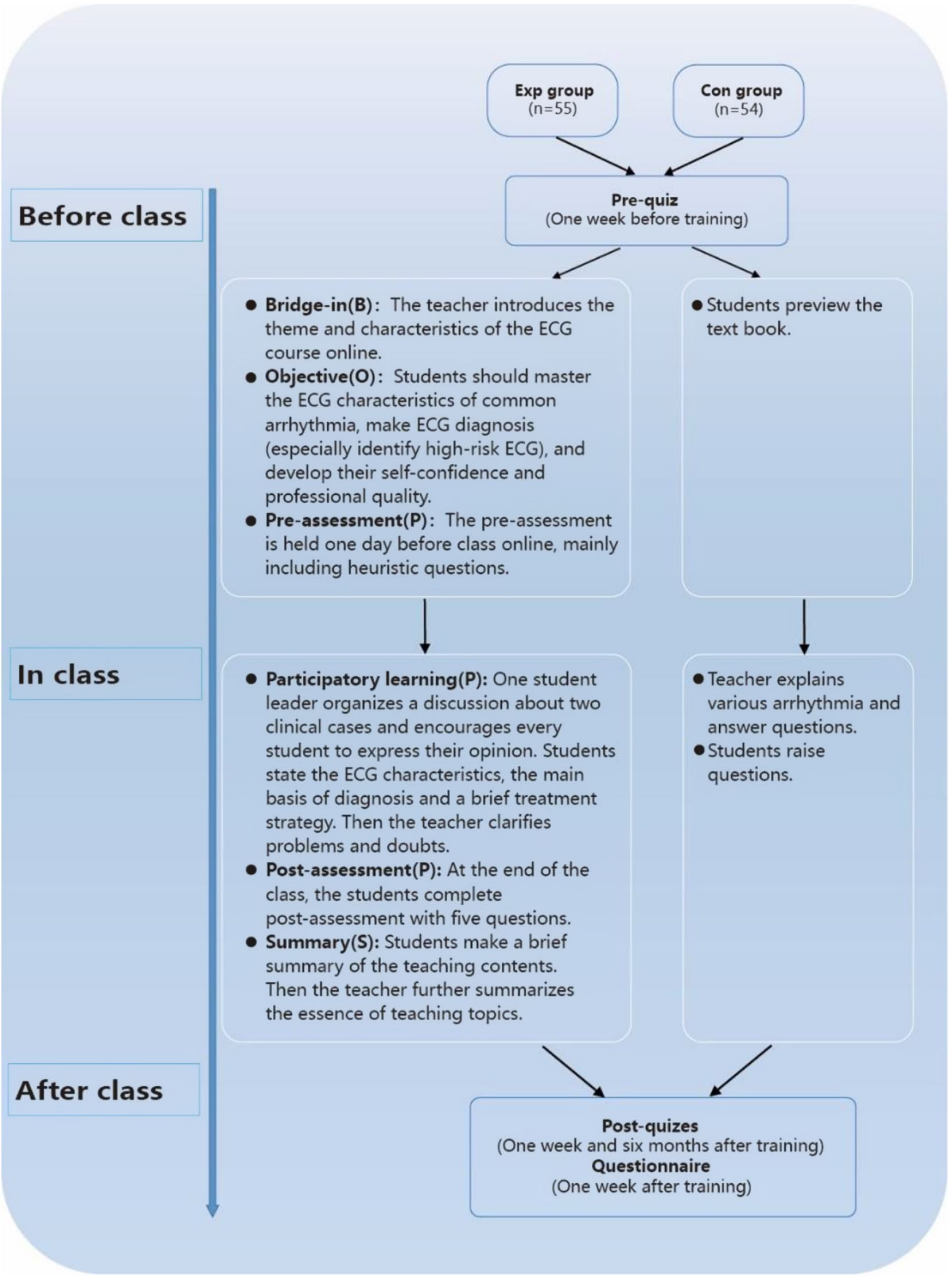
Flowchart of the study design. Exp group: experimental group with the combined BOPPPS and CBL method. Con: control group with the traditional LBL method

The BOPPPS instructional design is made up of six sections (Fig. 1). The specific steps of the BOPPPS-CBL model for ECG teaching are detailed below. To illustrate the application of these teaching approaches, we have chosen wide QRS complex tachycardia as an example in this study.

### Before class

**Bridge-in (B)** The teacher introduces the theme and characteristics of the ECG course online, with a focus on explaining the importance of the course and sparking students’ interest. For example, to attract students’ attention, we highlighted the cases of two celebrities who experienced sudden cardiac death. We then provided two anonymized ECGs of patients with wide QRS complex tachycardia, collected from clinical practice, ensuring that the examples were consistent with the syllabus.

**Objectives (O)** The teaching objectives must be clearly defined, ensuring that they are both achievable for students and evaluable for teachers. These three-level teaching objectives encompass cognitive, skill, and emotional goals. Specifically, students should master the ECG characteristics of common arrhythmias, make accurate ECG diagnoses (especially identifying high-risk ECGs), and work on developing their self-confidence and professional qualities. In the context of wide QRS complex tachycardia, the goals extend to students being able to make the correct ECG diagnosis and perform a differential diagnosis.

**Pre-assessment (P)** The pre-assessment was conducted online one day before class and primarily consisted of heuristic questions. This assessment serves to reveal the students’ levels of cognition and ECG interpretation abilities, thereby enabling teachers to flexibly adjust the depth and progress of the teaching content during class. Specific questions about wide QRS complex tachycardia for the students included:

1. What are the ECG characteristics of wide QRS complex tachycardia?
2. Can you identify high-risk ECGs of wide QRS complex tachycardia?

### In class

**Participatory learning (P)** This section represents the core of the BOPPPS-CBL model, with students taking the lead. The details are as follows:

1. Two real-world cases of wide QRS complex tachycardia were selected for students, and related questions were designed in line with the requirements of clinical application.
2. A student leader was assigned to organize a discussion, proposing questions and encouraging each student to express their opinion. Students could then seek answers to these questions from textbooks or other references.
3. Students described the ECG characteristics of wide QRS complex tachycardia, the main basis for diagnosis, and outlined a brief treatment strategy.
4. The teacher addressed questions and clarified any doubts that arose during the discussion, emphasizing the distinguishing features between similar cases.

**Post-assessment (P)** At the end of the class, the students completed a post-assessment consisting of five questions. These questions encompassed both basic theoretical knowledge and clinical case analysis. Timely assessment not only assists teachers in tracking the effectiveness of their teaching and optimizing their lesson plans, but it also helps students evaluate their mastery of the subject matter.

**Summary (S)** Students were encouraged to create a brief summary of the teaching contents. Following that, the teacher further distilled the essence of the teaching topics, highlighting the key points and identifying the difficulties within the teaching cases.

### LBL method for ECG course

The teacher distributed reference materials prior to class, including the recommended textbook. During the course, the teacher delivered a lecture to explain various arrhythmia (including ECG characteristics and treatment) followed by asking and answering questions (Fig. 1).

### Evaluation

The basic data of the nursing students, including age, gender, hospital level, education level, and experience in ECG interpretation after graduation, were collected one week before ECG training began. To assess the students’ understanding and application of knowledge, both groups underwent the same examinations, which consisted of one pre-quiz and two post-quizzes. These quizzes were administered one week before training, then one week and six months after training, respectively. All questions in the quizzes were divided into two categories: basic theoretical knowledge (40 points) and ECG interpretation (60 points). The theoretical knowledge section included 20 multiple-choice questions related to arrhythmia, each worth 2 points. The ECG interpretation section, on the other hand, consisted of 20 ECGs of arrhythmia, with 3 points awarded for each correct answer.

The questions in the three quizzes were evaluated by two different teachers to ensure consistency in difficulty levels. The total score for each quiz was 100 points, and the test time was 60 min. Afterward, the total score, as well as the scores for the basic theoretical knowledge and ECG interpretation sections, were calculated for each student.

To assess the students’ evaluation of learning attitudes and effects, a questionnaire survey was adopted at the end of the course, including self-learning enthusiasm, an increase of study load, systematization of teaching content, understanding of teaching content, student-teacher interaction, satisfaction of teaching mode, satisfaction of teaching effectiveness, self-confidence and interest in learning about ECGs. The 5-level Likert scoring method was adopted for each question, with 5 points for very satisfied/strongly agreed, 4 points for satisfied/agreed, 3 points for neutral, 2 points for dissatisfied/disagreed and 1 point for very dissatisfied/strongly disagreed.

### Statistical analysis

The normality of continuous data was assessed using the Shapiro-Wilk test. Depending on the data distribution, the results were presented as mean ± standard deviations (SDs) or median values, along with the interquartile range (IQR). Ages were compared using the Mann-Whitney U test, while categorical variables were analyzed with the chi-square test. ECG test scores of the two groups at different time points were evaluated through a general linear model for repeated measurements. For the 5-level Likert scores used in the students’ evaluation of learning attitudes and effects, the Mann-Whitney U test was also applied. All statistical analyses were conducted using SPSS 21.0 (SPSS Inc., Chicago, USA), with all tests being two-tailed, and significance set at P < 0.05.

## Results

### Demographic information of participants

A total of 109 nursing students were enrolled in this study and were randomly divided into two groups. The experimental group consisted of 55 nursing students and utilized a combined BOPPPS and CBL method, while the control group consisted of 54 nursing students and used the LBL method. Upon comparison of factors such as age, gender, hospital level, education level, and experience in ECG interpretation after graduation, no significant differences were found between these two groups (P > 0.05; Table 1).

**Table 1:** Baseline characteristics of students

Characteristic	Experimental Group (*n* = 55)	Control Group (*n* = 54)	*χ2*/*t*	*P*
**Age (Yrs.)**	24(22–26)	24(22–26)	0.132	0.895^a^
**Gender**			0.191	1.000^b^
Male	3	2		
Female	52	52		
**Hospital level**			0.261	0.696^b^
Class III hospital	23	20		
Class II hospital	32	34		
**Education level**			0.084	0.812^b^
University	45	43		
vocational high school	10	11		
**Experience in** **ECG interpretation**			0.373	0.658^b^
0 ~ 2 years	40	42		
> 2 years	15	12		

Experimental Group: experimental group with the combined BOPPPS and CBL method. Control Group: control group with the traditional LBL method. ^a^ The two groups were compared using the Mann-Whitney U test. ^b^ The two groups were compared using the chi-square test

### Comparison of ECG test scores

**Fig. 2:**
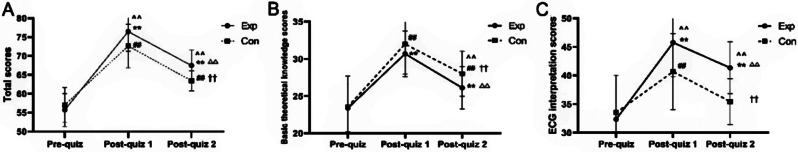
ECG test scores of Exp (n = 55) and Con (n = 54) groups at different time points. Exp: experimental group with the combined BOPPPS and CBL method. Con: control group with the traditional LBL method. Different time points: Pre-quiz: 1-week pre training; post-quiz 1: 1 week after training; post-quiz 2: 6 months after training. **A** total score; **B** theoretical score; **C** application score. Data are presented as mean ± standard deviation (SD); **p < 0.01 vs. Pre-quiz in Exp, ##p < 0.01 vs. Pre-quiz in Con, △△p < 0.01 vs. post-quiz 1 in Exp, †† p < 0.01 vs. post-quiz 1 in Con, ^^p < 0.01 vs. Con at different time points

We began by comparing the pre-and post-training quiz scores of the two groups (Fig. 2). One week before ECG training, the students were assessed with a pre-quiz. In the experimental group, the mean total score was 55.73 ± 4.35, and the theoretical knowledge and application scores were 23.38 ± 3.19 and 32.35 ± 5.25, respectively. For the control group, these scores were 57.06 ± 4.59, 23.56 ± 4.12 and 33.50 ± 6.56, respectively. There were no significant differences between the experimental and control groups (P > 0.05), indicating that the baseline between these two groups was comparable.

Following training, there were marked increases in the experimental group’s mean total score, theoretical knowledge, and application scores: from 55.73 to 76.45, 23.38 to 30.69, and 32.35 to 45.76, respectively (P < 0.01). Likewise, in the traditional group, these values increased from 57.06 to 72.67, 23.56 to 32.00, and 33.50 to 40.67, respectively (P < 0.01). Further analysis revealed that the experimental group’s mean total and application scores were significantly higher than those of the control group after training (76.45 ± 5.13 vs. 72.67 ± 5.78, 45.76 ± 5.92 vs. 40.67 ± 6.61, P < 0.01). However, no statistically significant difference was found in the theoretical knowledge scores between the two groups after training (30.69 ± 3.08 vs. 32.00 ± 4.00, P > 0.05).

To gauge retention of ECG theoretical knowledge and interpretation skill, a second post-quiz was conducted six months after training. Although the mean total scores, theoretical knowledge, and application scores of both groups significantly decreased, the experimental group’s mean total and application scores were still significantly higher than those of the control group (67.45 ± 4.08 vs. 63.44 ± 2.65, 41.35 ± 4.53 vs. 35.44 ± 4.04, P < 0.01). Interestingly, the experimental group’s theoretical knowledge score was significantly lower than the control group’s (26.11 ± 2.86 vs. 28.00 ± 3.01, P < 0.01).

Moreover, when compared with the pre-quiz, both groups still showed a significant improvement in the mean total score six months after training (P < 0.01). The experimental group’s improvement was seen in both theoretical and application scores (P < 0.01). In contrast, the control group’s most significant improvement was in theoretical score (P < 0.01), with no noticeable advancement in application score (P > 0.05) (Fig. 2).

### Comparison of students’ attitudes

**Fig. 3:**
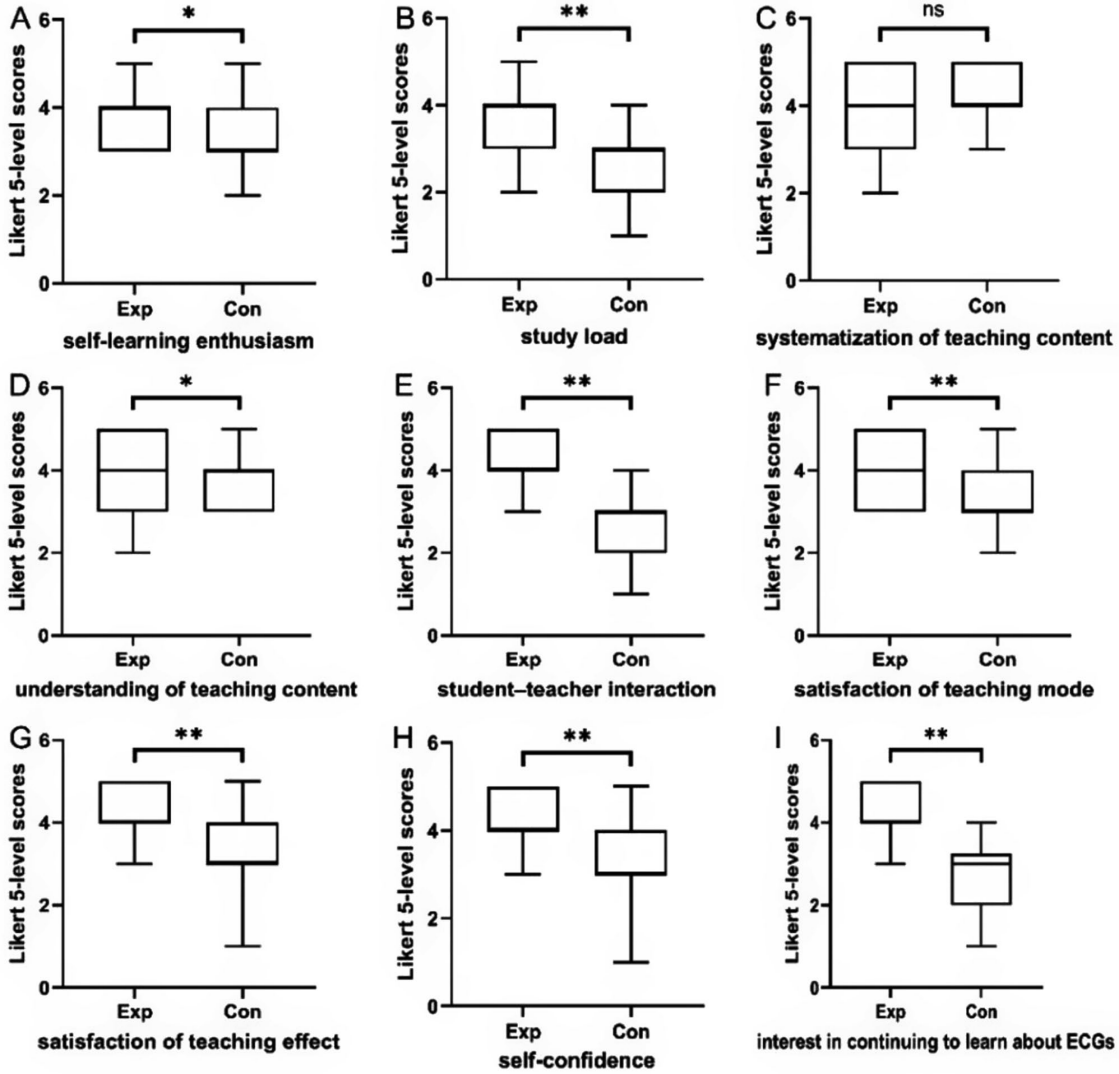
Five-level likert scores of students’ attitudes in Exp (n = 55) and Con (n = 54) groups. Exp: experimental group with the combined BOPPPS and CBL method. Con: control group with the traditional LBL method. **A** self-learning enthusiasm; **B** study load; **C** systematization of teaching content; **D** understanding of teaching content; **E** student-teacher interaction; **F** satisfaction of teaching mode; **G** satisfaction of teaching effect; H self-confidence; **I** interest in continuing to learn about ECGs. NS: no significant difference, *p < 0.05 Exp vs. Con, **p < 0.01 Exp vs. Con

A total of 109 questionnaires were sent out, all of which were recovered, resulting in a recovery rate of 100%. When comparing the experimental group with the control group, significant improvements were observed in the experimental group in various aspects. These included self-learning enthusiasm, understanding of teaching content, student-teacher interaction, satisfaction with teaching mode, satisfaction with teaching effectiveness, self-confidence, and interest in learning ECG, all of which were significantly higher (P < 0.05 for the first two aspects and P < 0.01 for the rest). However, the systematization of teaching content was found to be comparable between these two groups (P > 0.05). Additionally, it is worth noting that the majority of students in the experimental group indicated an increase in study load (P < 0.01) (Fig. 3).

## Discussion

Effective ECG education for nurses is crucial, as it necessitates both a strong foundational understanding and sharp analytical skills. However, traditional lecture-based learning (LBL) often falls short in fostering active engagement and enhancing ECG competency, which is pivotal in diagnosing arrhythmias and coronary syndromes [19, 20]. Students frequently exhibit gaps in basic knowledge and a reluctance to apply theory confidently [19, 20]. To bridge this divide, educational experts advocate for active and self-directed learning strategies to facilitate better knowledge retention and complex problem-solving abilities, thus improving overall outcomes of diagnosis and therapy [21–23].

A notable advancement in teaching methodologies is the BOPPPS model, which emphasizes student participation and consistent feedback to boost instructional effectiveness [24, 25]. This model garners preference among students, leading to increased satisfaction, engagement, and the honing of critical thinking skills [24, 25]. The systematic nature of the framework suggests that it has potential to be preferred for teaching ECG interpretation to nurse trainees. Moreover, the Case-Based Learning (CBL) strategy, pioneered by Harvard Medical School, employs real-life scenarios to sharpen medical students’ academic prowess and case analysis capabilities [26]. By incorporating actual clinical data, including medical histories and laboratory findings, CBL not only enhances knowledge and skills but also cultivates clinical reasoning. Its interactive aspect further boosts student involvement and the enjoyment of the learning process [27].

Mastering ECG interpretation is challenging, and no single instructional method can guarantee maximum proficiency for all learners. With this in mind, we have evaluated whether the BOPPPS-CBL model can effectively enhance the teaching effect of ECG interpretation. Our study demonstrates that the implementation of the BOPPPS-CBL model yielded superior total test scores for nursing students in the experimental group compared to the control group.

According to Bloom’s Taxonomy, a conceptual framework used to classify educational objectives originating from the work of Benjamin Bloom in 1956 [28]. This taxonomy classifies cognitive activities into six hierarchical levels: remembering, understanding, applying, analyzing, evaluating, and creating. In our study, the test consisted of two parts, each focusing on different aspects: basic theoretical knowledge and ECG interpretation capability. The assessment of remembering and understanding was evaluated by the test of basic theoretical knowledge, while the assessment of higher-level cognition in Bloom’s taxonomy (application and analysis) was performed through the ECG interpretation test. We further dissected the overall improvement structure to identify the dominant part in score improvements from pre- to post-quiz. There was a significant increase in ECG interpretation capability after the experimental group’s training. Our studies have shown that experimental teaching is more effective in helping students master basic knowledge and apply it to interpret ECG. These findings echo a prior study that observed elevated levels of problem-solving skills and analytical skills in the BOPPPS-CBL model group compared to the traditional teaching group [8].

Interestingly, regardless of the teaching method used, scores in basic knowledge improved significantly after training in both groups, with no significant difference between them. Moreover, our study revealed significant differences in students’ self-learning enthusiasm and course satisfaction between the two groups, supporting the findings of previous studies [8, 29]. We also examined the BOPPPS-CBL model’s potential to facilitate long-term retention of basic theoretical knowledge and ECG interpretation. After six months, though ECG quiz scores decreased in both groups, the experimental group consistently outperformed the control group. This indicates that the BOPPPS-CBL model’s teaching effectiveness had a more lasting impact on students’ ECG interpretation abilities.

Our new BOPPPS-CBL model approach offers several advantages over traditional teaching methods. The model positively affects both class goal achievement and the impact of teaching modes on learning. Its “bridge-in” stage can spark students’ interest through thought-provoking questions, such as the significance of ECG for patients with severe cardiovascular diseases. This intriguing introduction can capture students’ enthusiasm, helping them see the importance of ECG in medical practice. Further stages emphasize learning objectives, transform the classroom dynamic from teacher-centered to student-centered, and facilitate a multi-interactive learning process. During the BOPPPS-CBL teaching, clinical cases encourage students to think independently, analyze data, and make clinical diagnoses and decisions [30]. It’s an effective method, promoting active learning and developing clinical reasoning skills. Consequently, the BOPPPS-CBL model in ECG teaching not only improves students’ interest in learning but also better trains their abilities in clinical case analysis. ECG teaching demands students comprehend abstract knowledge and theory, subsequently linking them to the capacity for ECG interpretation. Though understanding these basics is crucial, especially for nurses in cardiology departments, the true challenge lies in teaching students to apply this knowledge to real-world problems. Developing an effective model like BOPPPS-CBL can substantially enhance the teaching effect in ECG instruction.

In summary, the BOPPPS model is a student-oriented, teacher-led interactive method that emphasizes clear learning objectives and active student participation. Combined with CBL teaching, which is based on typical clinical cases, it encourages students to think independently and make clinical decisions. The BOPPPS-CBL approach in ECG teaching is a comprehensive and effective way to foster positive attitudes towards teaching and learning.

### Limitations

This study is subject to three primary limitations. Firstly, the scope of the investigation was confined to trainee nurses; therefore, subsequent studies incorporating a broader range of participants are essential to corroborate the efficacy of the combined method proposed. Secondly, the limited sample size of this study necessitates further research with an expanded cohort to thoroughly assess the method’s impact. Thirdly, the longitudinal effects of this innovative approach were not examined adequately; future studies should employ multi-center randomized controlled trials with extended follow-up durations to evaluate the sustained effectiveness of the method.

## Conclusion

Our study is the first to combine the BOPPPS model with the CBL method in the ECG course for nursing students. We have found that this combination is an effective and innovative approach, improving nurses’ capabilities in ECG interpretation and diagnosis. Further research is required to minimize potential biases in our findings. Additionally, more studies should be conducted to determine whether this method can be applied to other groups, such as healthcare professionals, medical students, and public health students.

## Data Availability

Please contact the corresponding author for data availability.

## References

[1] Miranda DF, Lobo AS, Walsh B, Sandoval Y, Smith SW (2018). New insights into the Use of the 12-Lead Electrocardiogram for diagnosing Acute Myocardial Infarction in the Emergency Department. Can J Cardiol.

[2] Chang A, Cadaret LM, Liu K (2020). Machine learning in Electrocardiography and Echocardiography: Technological advances in clinical cardiology. Curr Cardiol Rep.

[3] Siontis KC, Noseworthy PA, Attia ZI, Friedman PA (2021). Artificial intelligence-enhanced electrocardiography in Cardiovascular Disease management. Nat Rev Cardiol.

[4] Kim S, Kim CG (2020). Effects of an Electrocardiography Training Program: Team-based learning for early-stage Intensive Care Unit nurses. J Contin Educ Nurs.

[5] Rui Z, Lian-Rui X, Rong-Zheng Y, Jing Z, Xue-Hong W, Chuan Z (2017). Friend or foe? Flipped Classroom for Undergraduate Electrocardiogram Learning: a Randomized Controlled Study. BMC Med Educ.

[6] Alhazmi A, Quadri MFA (2020). Comparing case-based and lecture-based learning strategies for orthodontic case diagnosis: a randomized controlled trial. J Dent Educ.

[7] Gao J, Yang L, Zhao J, Wang L, Zou J, Wang C, Fan X (2020). Comparison of problem-based learning and traditional teaching methods in medical psychology education in China: a systematic review and meta-analysis. PLoS ONE.

[8] Chen L, Tang XJ, Chen XK, Ke N, Liu Q (2022). Effect of the BOPPPS model combined with case-based learning versus lecture-based learning on ophthalmology education for five-year paediatric undergraduates in Southwest China. BMC Med Educ.

[9] Li Z, Cai X, Zhou K, Qin J, Zhang J, Yang Q, Yan F (2023). Effects of BOPPPS combined with TBL in surgical nursing for nursing undergraduates: a mixed-method study. BMC Nurs.

[10] Li S, Liu Q, Guo S, Li Y, Chen F, Wang C, Wang M, Liu J, Liu X, Wang D (2023). Research on the application of the blended BOPPPS based on an online and offline mixed teaching model in the course of fermentation engineering in applied universities. Biochem Mol Biol Educ.

[11] Ma X, Ma X, Li L, Luo X, Zhang H, Liu Y (2021). Effect of blended learning with BOPPPS model on Chinese student outcomes and perceptions in an introduction course of health services management. Adv Physiol Educ.

[12] Williams B (2005). Case based learning–a review of the literature: is there scope for this educational paradigm in prehospital education?. Emerg Med J.

[13] Novack JP (2020). Designing cases for case-based immunology teaching in large medical school classes. Front Immunol.

[14] Nair SP, Shah T, Seth S, Pandit N, Shah GV (2013). Case based learning: a method for better understanding of biochemistry in medical students. J Clin Diagn Res.

[15] Cen XY, Hua Y, Niu S, Yu T (2021). Application of case-based learning in medical student education: a meta-analysis. Eur Rev Med Pharmacol Sci.

[16] McLean SF. Case-based learning and its application in medical and health-care fields: a review of worldwide literature. J Med Educ Curric Dev. 2016;3. 10.4137/JMECD.S20377 PMC5736264 29349306

[17] Krupat E, Richards JB, Sullivan AM, Fleenor TJ, Schwartzstein RM (2016). Assessing the effectiveness of case-based collaborative learning via Randomized Controlled Trial. Acad Med.

[18] Fent G, Gosai J, Purva M (2015). Teaching the interpretation of electrocardiograms: which method is best?. J Electrocardiol.

[19] Zeng HL, Chen DX, Li Q, Wang XY (2020). Effects of seminar teaching method versus lecture-based learning in medical education: a meta-analysis of randomized controlled trials. Med Teach.

[20] Cikrikci Isik G, Safak T, Tandogan M, Cevik Y (2020). Effectiveness of the CRISP Method on the primary Cardiac Arrhythmia Interpretation Accuracy of nurses. J Contin Educ Nurs.

[21] Paul S (2001). Understanding advanced concepts in atrioventricular block. Crit Care Nurse.

[22] Hernandez JM, Glembocki MM, McCoy MA (2019). Increasing nursing knowledge of ST-Elevated Myocardial Infarction recognition on 12-Lead electrocardiograms to improve patient outcomes. J Contin Educ Nurs.

[23] Pothitakis C, Ekmektzoglou KA, Piagkou M, Karatzas T, Xanthos T (2011). Nursing role in monitoring during cardiopulmonary resuscitation and in the peri-arrest period: a review. Heart Lung.

[24] Yang Y, You J, Wu J, Hu C, Shao L (2019). The Effect of Microteaching combined with the BOPPPS Model on Dental materials Education for Predoctoral Dental Students. J Dent Educ.

[25] Hu K, Ma RJ, Ma C, Zheng QK, Sun ZG (2022). Comparison of the BOPPPS model and traditional instructional approaches in thoracic Surgery education. BMC Med Educ.

[26] Gholami M, Changaee F, Karami K, Shahsavaripour Z, Veiskaramian A, Birjandi M (2021). Effects of multiepisode case-based learning (CBL) on problem-solving ability and learning motivation of nursing students in an emergency care course. J Prof Nurs.

[27] Kassebaum DK, Averbach RE, Fryer GE (1991). Student preference for a case-based vs. lecture instructional format. J Dent Educ.

[28] Nascimento J, Siqueira TV, Oliveira JLG, Alves MG, Regino D, Dalri MCB (2021). Development of clinical competence in nursing in simulation: the perspective of Bloom’s taxonomy. Rev Bras Enferm.

[29] Xu Z, Che X, Yang X, Wang X (2023). Application of the hybrid BOPPPS teaching model in clinical internships in gynecology. BMC Med Educ.

[30] Harman T, Bertrand B, Greer A, Pettus A, Jennings J, Wall-Bassett E, Babatunde OT (2015). Case-based learning facilitates critical thinking in undergraduate nutrition education: students describe the big picture. J Acad Nutr Diet.

